# Supplementary material for: Learning curve and surgical outcome of robotic assisted colorectal surgery with ERAS program
Source: Sci Rep. 2022 Nov 29;12:20566. doi: 10.1038/s41598-022-24665-w (PMC9709162; doi:10.1038/s41598-022-24665-w)
Supplement: Supplementary file 1 — Supplementary Information 1. [file 41598_2022_24665_MOESM1_ESM.zip › LSC Raw data-Ñ[▒K/094/N_ñw╝╨░O▒Kñσ.pdf]

W55 / W55

臺中榮民總醫院
手術室護理記錄

[redacted]

基本資料

- 室別： 二醫手術室-31室，手術科別： CRS，手術類別： 常規，日期： 2020/09/24
- 進入時間： 1100 ； 麻醉開始時間： 1105 ； Time Out時間： 1206 ； 劃刀時間： 1207 ； 手術結束時間： 1730 送出時間： 1800 ；
- 麻醉方式： GE
  診斷：T COLON CA SP LOOP T COLOSTOMY, GBS
  手術名稱：1.Laparoscopic right colectomy and anastomosis2.Closure of ileostomy3.Laparoscopic cholecystectomy
  手術局部用藥：nil
  術前預防性抗生素：
    - 抗生素名稱一： Veterin 1GM IVD(由麻醉科護理人員給予)
    - 時間： 1205
    - 上次簽章: 謝昀安
  ，追加預防性抗生素：
    - 抗生素名稱一： Veterin 1GM IVD/由麻醉護理師給予
    - 時間： 1605
    - 上次簽章: 林宜旻
- 手術醫師： 蔣鋒帆，劉嘯天，陳映菁，林昌霖，楊捷儒
- 麻醉醫師： VS 徐煥
-
    - 刷手護士A ： 林宜旻，時間： 1100，接替刷手一： 卓汶珊，時間： 1200，接替刷手二： 吳雅華，時間： 1230
    - 流動護士A ： 吳雅華，時間： 1100，接替流動一： 謝昀安，時間： 1200，接替流動二： 林宜旻，時間： 1230

皮膚狀況

A-V Shunt： 無
術前： 完整
  記錄者： 上次簽章: 吳雅華
術後： 完整
  記錄者： 上次簽章: 林宜旻

手術準備

手術臥式 單一手術臥式

-
    - 手術臥式： 仰臥
    - 使用附件： 溫毯 38 ℃，矽膠枕頭，頭圈
    - 皮膚準備： 10%Aq Betadine，Hibiscrub，2% Chlorhexidine
    - 電燒： 使用電燒: 電極片位置： 其它： 迴路電極墊，記錄者： 上次簽章: 吳雅華

止血帶T： 無

植入物

- 植入物名稱/號碼/數量/部位：Surgiwrap 0.02x100x130mm/壹/pelvic

，記錄者：上次簽章: 林宜旻

標本送檢

- 細菌培養 厭氧，採取部位: 1. Gall bladder bile，數量： 壹
- 細胞學 無
- 冰凍切片 無
- 病理標本
  組織名稱一：A-T colon
  ，
  組織名稱二：Gall bladder

- 截肢 無

標本交付 標本： 貳 袋，標本： 零 盒，標本： 零 瓶，細胞學： 零 管，Culture Tube： 壹 管，被交付醫師： R 楊捷儒，

- 記錄者： 上次簽章: 林宜旻
- 時間： 1800

傷口術後敷料

傷口敷料：

- 請於圖上標示部位：

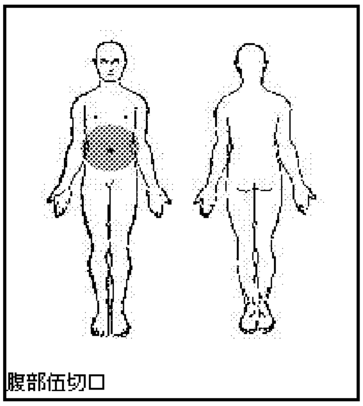

- 傷口敷料： Mepore

傷口敷料：

- 請於圖上標示部位：

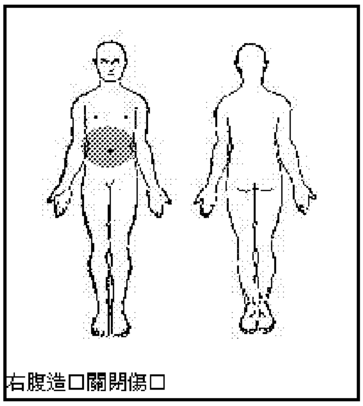

- 傷口敷料： 生理食鹽水濕紗壹塊(2x2白紗剪一半)+白紗覆蓋固定

導管

導管	天數	導管資訊	插入日期	插入時間	更換日期	拔除日期	拔除時間	拔除原因	預計拔除/更換	預計拔除/更換日期
A-line (1)	1	號碼:20 G; 部位:左手;	2020/09/24	1140						
Foley(1)	1	號碼:16 Fr.; 部位:膀胱;	2020/09/24	1200						

用物計數

- 手術中出血量： 100 c.c.
- 手術中尿量： 400 c.c.
-
    - 其它： 無

用物計數： 多切口同時縫合

- 1.器械： 正確，2.縫針： 正確，3.刀片： 正確，4.紗布： 正確，5.腹部墊： 未使用，6.紗球： 未使用，7.PEANUTS ： 未使用，8.紗條： 未使用，9.絲帶： 未使用，10.針頭： 未使用，11.Vessel loop： 未使用，12.Boots： 未使用，13.Jelco： 未使用，14.棉條： 未使用，
    - 第一次：
        - 刷手護士 ： 吳雅華
        - 流動護士： 上次簽章: 林宜旻
        - 時間： 1620

  ,
    - 第二次：
        - 刷手護士 ： 吳雅華
        - 流動護士： 上次簽章: 林宜旻
        - 時間： 1640

  ,
    - 第三次：
        - 刷手護士 ： 吳雅華
        - 流動護士： 上次簽章: 林宜旻
        - 時間： 1705

備註

備註：NIL

- 記錄者： 上次簽章: 林宜旻
- 時間： 1740

填寫護理人員 林宜旻

正式摘要

W55 / W55

doctorRank:B
